# Current update on the protective effect of naringin in inflammatory lung diseases

**DOI:** 10.17179/excli2022-4752

**Published:** 2022-03-07

**Authors:** Gaurav Gupta, Waleed Hassan Almalki, Imran Kazmi, Neeraj Kumar Fuloria, Shivkanya Fuloria, Vetriselvan Subramaniyan, Mahendran Sekar, Sachin Kumar Singh, Dinesh Kumar Chellappan, Kamal Dua

**Affiliations:** 1 School of Pharmacy, Suresh Gyan Vihar University, Jagatpura 302017, Mahal Road, Jaipur, India; 2 Department of Pharmacology, Saveetha Dental College, Saveetha Institute of Medical and Technical Sciences, Saveetha University, Chennai, India; 3 Department of Pharmacology, College of Pharmacy, Umm Al-Qura University, Makkah, Saudi Arabia; 4 Department of Biochemistry, Faculty of Science, King Abdulaziz University, Jeddah, Saudi Arabia; 5 Faculty of Pharmacy, AIMST University, Bedong 08100, Kedah, Malaysia; 6 Faculty of Medicine, Bioscience and Nursing, MAHSA University, Jalan SP 2, Bandar Saujana Putra, 42610 Jenjarom Selangor, Malaysia; 7 Department of Pharmaceutical Chemistry, Faculty of Pharmacy and Health Sciences, Royal College of Medicine Perak, Universiti Kuala Lumpur, Ipoh 30450, Perak, Malaysia; 8 School of Pharmaceutical Sciences, Lovely Professional University, Phagwara, Punjab, 144411, India; 9 Faculty of Health, Australian Research Center in Complementary and Integrative Medicine, University of Technology Sydney, Ultimo, NSW, 2007, Australia; 10 Department of Life Sciences, School of Pharmacy, International Medical University, Kuala Lumpur, 57000, Malaysia; 11 Discipline of Pharmacy, Graduate School of Health, University of Technology Sydney, NSW 2007, Australia

## ⁯

Understanding the role of inflammation in developing respiratory illnesses such as COPD, asthma, and lung cancer, is critical. Natural cures are regaining favor as effective treatments for various ailments (Heidary Moghaddam et al., 2020[14]). A flavanone glycoside named naringin (NAR) is found in aromatic Chinese herbal treatments and citrus fruits. Even though several biological and pharmacological properties of NAR have been found via study, only a few systematic reviews have been published (Wadhwa et al., 2021[29]). However, there is a scarcity of studies focusing on NAR's therapeutic potential in respiratory system inflammation. NAR's alleged anti-inflammatory properties influence many pro-inflammatory cytokines, including the NF-κB, ERK1/2, and p38 MAPK pathways in the pathophysiological processes associated with chronic respiratory disorders (Chen et al., 2016[8]). This review will be very useful to researchers in this field. It will take them in a new direction in their hunt for innovative medications to treat respiratory illnesses (Table 1(Tab. 1); References in Table 1: Ahmad et al., 2015[1]; Akintunde et al., 2020[2]; Ali et al., 2017[3]; Bear and Teel, 2000[4]; Cerkezkayabekir et al., 2017[5]; Chang et al., 2017[6]; Chen et al., 2013[9], 2014[10], 2018[7]; Clementi et al., 2021[11]; Fouad et al., 2016[12]; Guihua et al., 2016[13]; Hsiao et al., 2007[15]; Jiao et al., 2015[16]; Kim et al., 2018[17]; Liu et al., 2011[20], 2012[19], 2018[18]; Luo et al., 2012[21]; Nie et al., 2012[22]; Schwarz et al., 2005[23]; Seyedrezazadeh et al., 2015[24]; Shi et al., 2014[27], 2019[25], 2021[26]; Turgut et al., 2016[28]; Wang et al., 2016[30]; Wu et al., 2021[31]; Yao et al., 2021[32]; Zhang et al., 2013[33]).

| Inflammatory lung disease | Key findings | References |
|---|---|---|
| **Asthma** | Guihua et al. investigated the anti-inflammatory effects of naringin (NAR) against ovalbumin-induced airway inflammation using an asthma animal model. The GABA3 inhibitor naringin was shown to have a considerable effect on T-bet expression, indicating that naringin might be used to treat persons with allergic asthma. | Guihua et al., 2016 |
| | Jiao et al. employed an ovalbumin-induced cough-variant asthma animal model to investigate the therapeutic advantages of NAR. Ovalbumin-induced cough and airway hyperresponsiveness (AHR) was significantly reduced when NAR was given consecutively compared to the model group. In addition, leukocytes, IL-4, IL-5, and IL-13 levels in BALF decreased considerably. The impact of naringin treatment on lung tissue pathology was also noticeable. According to these data, people with cough-variant asthma (CVA) may benefit from NAR. | Jiao et al., 2015 |
| | Wang et al. discovered that the naturally occurring chemical naringin relaxes smooth muscle cells in the airways *in vivo* and *in vitro* models of ovalbumin-induced asthma. When methacholine was administered to normal Balb/c mice, and asthmatic Balb/c mice provoked with ovalbumin, NAR (15, 30 and 60 µg) reduced bronchial airway resistance. To summarize, the findings presented here indicate that NAR can relax murine ASMCs, indicating that it is a pharmacological agent worth further exploration to develop future asthma-treating bronchodilators. | Wang et al., 2016 |
| | Seyedrezazadeh et al. investigated whether flavanones, grapefruit, orange juices, and hesperetin-NAR may reduce airway inflammation and remodeling in a chronic asthma model. The researchers observed that hesperetin and NAR were more efficient than orange or grapefruit juice in decreasing airway structural remodeling in a rat model of HDM-induced asthma. | Seyedrezazadeh et al., 2015 |
| | Shi et al. investigated the effects of naringenin on allergic airway remodeling in mice. After treatment with NAR, the levels of Th2 cytokines in the BALF were dramatically decreased. Consequently, NAR might be used to treat asthma by inhibiting the remodeling of the airways. | Shi et al., 2014 |
| **COPD** | In a guinea pig model, Luo et al. discovered that NAR reduces the severity of chronic bronchitis symptoms such as coughing up blood, hyperresponsiveness of the airways, and inflammation of the airways. In addition, NAR contains antitussive, anti-AHR, and anti-inflammatory properties, according to a study on guinea pigs with chronic CS-induced bronchitis, suggesting that it has unique therapeutic potential for treating chronic bronchitis. | Luo et al., 2012 |

EXCLI Journal 2022;21:573-579 – ISSN 1611-2156
Received: February 08, 2022, accepted: February 22, 2022, published: March 07, 2022

| Inflammatory lung disease | Key findings | References |
|---|---|---|
| | The effects of NAR on COPD induced by cigarette smoke were investigated by Liu et al. using A549 cells and BALB/c mice (CS). NAR has been demonstrated to significantly impact lung function and the production of pro-inflammatory cytokines, including TNF-α and MMP-9, in the blood and serum of the CS animal group. | Liu et al., 2018 |
| **Lung Cancer** | According to Chen et al., NAR controls miR-126/VCAM-1 in NSCLC. NAR has anti-cancer capabilities by inhibiting NSCLC through the miR-126/VCAM-1 signaling pathway. | Chen et al., 2018 |
| | According to Kim and colleagues, NAR protects mice against acrolein-induced lung damage via regulating apoptotic and inflammatory signaling pathways. In addition, NAR provided lung protection in mice against the environmental toxin acrolein via modulating the MAPK/p53/NF-κB signaling pathways. | Kim et al., 2018 |
| | As per Hsiao et al., six flavanones were shown to have anti-metastatic effects on lung cancer cell lines, including NAR, 4-OH flavanone, 2-OH flavanone, flavanone, and naringenin. The capacity of naringin to suppress NAR oral therapy demonstrated its potential to prevent A549 and Lewis lung cancer (LLC) cell *metastasis in vivo*. NAR inhibits the capacity of lung cancer cells to infiltrate and disseminate, making it an adjuvant treatment for metastasis prevention. | Hsiao et al., 2007 |
| | Chang et al. experimented with learning more about how NAR influences the migration of A549 lung cancer cells. They discovered that NAR inhibited the action of AKT and reduced MMP-9 and MMP-2, among other things. | Chang et al., 2017 |
| | Shi et al. investigated the anti-proliferative and apoptotic NAR's impact on human lung carcinoma cells. Ingrown human cancer cells, naringenin at concentrations of 100 and 200 mol/L significantly decreased the extent of wounds compared to untreated lung cancer cells. In addition, the expression of caspase-3 was increased, but NAR reduced the expression of MMP-2 and MMP-9. According to these studies, NAR seems to be a successful therapy for lung cancer in people. | Shi et al., 2021 |
| **Pulmonary Fibrosis** | Turgut et al. discovered that NAR protects Wistar rats against bleomycin-induced fibrosis of the lungs. The antioxidant capabilities of NAR contribute to this positive effect by reducing the levels of pro-inflammatory cytokines avoiding or eliminating oxygen free radicals from the medium. | Turgut et al., 2016 |
| | According to Chen et al., researchers discovered that NAR could protect mice's lungs against acute lung damage and fibrosis caused by paraquat (PQ). According to the results, naringin (60 and 120 mg/kg) substantially decreased TIMP-1, MMP-9, and TNF-α induced upregulations and pulmonary malonaldehyde hydroxyproline levels and lung fibrosis deposition while improving HO-1, GSH-Px and SOD activities. According to these findings, NAR protected against ALI and lung fibrosis caused by PQ. | Chen et al., 2013 |
| **Lung Injury** | The anti-inflammatory activities of NAR were investigated in mice with acute lung damage caused by LPS. According to the findings, NAR reduces inflammation in LPS-induced ALI mice by lowering iNOS and MPO, pulmonary neutrophil infiltration and TNF-α through NF-κB blocking. | Liu et al., 2011 |

EXCLI Journal 2022;21:573-579 – ISSN 1611-2156
Received: February 08, 2022, accepted: February 22, 2022, published: March 07, 2022

| Inflammatory lung disease | Key findings | References |
|---|---|---|
| | Chen et al. investigated the effects of NAR on acute lung injury (ALI) produced by LPS in mice and beagle dogs. Nitric oxide (NO) is a strong mucolytic agent. NAR was shown to have various mucoactive effects in this research, including a reduction in goblet cell hyperplasia and increased sputum output. | Chen et al., 2014 |
| | Cerkezkayabekir et al. investigated the impact of NAR on the small intestine, liver, kidneys, and lungs during gut ischemia/reperfusion injury. According to the results, NAR had a significant, favorable effect on the biochemical parameters of I/R by reducing NO levels, balancing iNOS and eNOS expression, and decreasing arginase activity. | Cerkezkayabekir et al., 2017 |
| | Liu et al. aimed to evaluate the effect of naringin on chemokine expression in LPS-induced RAW 264.7 macrophages and to provide insights into the possible underlying mechanisms. The finding demonstrates that naringin reduces IL-8, MCP-1 and MIP-1α secretion and mRNA expression, possibly by blocking the activation of the NF-κB and MAPK signaling pathways in LPS-induced RAW 264.7 macrophages. | Liu et al., 2012 |
| | Nie et al. discovered that NAR might diminish chronic pulmonary neutrophilic inflammation in rats exposed to cigarette smoke. Using NAR (20, 40 and 80 mg/kg), neutrophils were halted from infiltrating, MPO and MMP-9 activities were reduced, and IL-8 release was repressed. In rats infected with *C. difficile*, the anti-inflammatory drug NAR has been demonstrated to diminish persistent lung neutrophilic inflammation (CS). | Nie et al., 2012 |
| | Shi et al. studied the effects of DPM on ASL secretion and the involvement of NAR in this process to determine NAR's therapeutic potential for treating abnormal respiratory secretion caused by PM2.5. DPM lowered ASL secretion and increased the viscosity of the liquid, according to *in vitro* and *in vivo* investigations. Furthermore, NAR has been shown to protect cells against DPM-induced damage, boost CFTR, AQP1, and AQP5 mRNA and protein expression, regulate apical CFTR insertion favorably, and promote CFTR activation by raising intracellular cAMP. These findings suggested that NAR had a role in regulating DPM-induced unusual respiratory discharge. | Shi et al., 2019 |
| | According to Ahmed and colleagues, NAR has been found to exhibit anti-inflammatory properties in a mouse model of carrageenan-induced pleurisy. NAR inhibited the production of Th1 and Th2 cytokines in the pleural fluid. Furthermore, NAR boosted I-B degradation in lung tissue while decreasing NF-κB activation. As a result, NAR treatment may minimize tissue damage by lowering pro-inflammatory and anti-inflammatory mediator production in lung tissue. | Ahmad et al., 2015 |
| | Wu et al. discovered that NAR inhibits the ERK and NF-κB signaling pathways and the progression of EndMT in the pulmonary arteries, which may help alleviate pulmonary arterial hypertension. | Wu et al., 2021 |
| | Bear et al. investigated the *in vitro* metabolism of NNK and the dealkylation of methoxyresorufin (MROD) and pentoxyresorufin (PROD) in Syrian golden hamster liver and lung microsomes. Citrus fruits' naringenin and quercetin may block NNK-activating cytochrome P450 (CYP) isoforms, potentially protecting against NNK-induced carcinogenesis. | Bear and Teel, 2000 |

*EXCLI Journal 2022;21:573-579 – ISSN 1611-2156*
*Received: February 08, 2022, accepted: February 22, 2022, published: March 07, 2022*

| Inflammatory lung disease | Key findings | References |
|---|---|---|
| | Akintunde et al. studied NAR, a naturally occurring flavonoid, for its possible oculoprotective and lung-protective effects, in a rat model of wood smoke (WS)-induced toxicity. NAR injections may increase the activity of antioxidant enzymes in the eyes and lungs while lowering AChE and nitric oxide levels in the lungs and eyes, the main organs exposed to WS. Furthermore, the data suggest that individuals who have been exposed to PM10 WS may benefit from NAR therapy. | Akintunde et al., 2020 |
| | Fouad et al. investigated naringenin to determine whether it might protect rats against ALI produced by LPS. NAR's anti-inflammatory, antioxidant, antinitrosative, and antiapoptotic characteristics significantly protected rats against ALI caused by LPS. | Fouad et al., 2016 |
| | Ali et al. investigated NAR's capacity to protect against the negative effects of B[a]P. According to these findings, naringenin supplementation protects alveoli and epithelium from oxidative stress and lung damage caused by B[a]P by maintaining alveolar and epithelial integrity. | Ali et al., 2017 |
| | Schwarz et al. investigated the inhibitory effects of quercetin and NAR on the final bioactivation process of benzo[a]pyrene (B[a]P), an important family of lung carcinogens. Those with wild-type CYP1A1 genes, according to our results, may benefit more from quercetin's chemopreventive qualities than those with variant CYP1A1 genes. | Schwarz et al., 2005 |
| Pneumonia | According to Yao and colleagues, NAR may effectively cure pediatric bronchial pneumonia. This medicine reduced bronchial pneumonia complications and their negative consequences, controlled inflammation, and shortened the time it took for clinical signs to go. | Yao et al., 2021 |
| | Zhang et al. study NAR's influence on toxin production and therapeutic efficacy in treating staphylococcal pneumonia. Using *S. aureus pneumonia*, scientists observed that NAR decreased lung damage and inflammation. | Zhang et al., 2013 |
| COVID-19 | Clementi et al. discovered that naringenin is an effective anti-CoV pharmacological technique, and their findings indicate the tremendous potential for effective and safe prevention and therapy. Another reason to employ hydrophobic compounds like naringenin as a possible antiviral treatment is that it is a selective inhibitor of Two-Pore Channels, which may pass through biological membranes and reach intracellular compartments (TPCs). | Clementi et al., 2021 |

## *Conflict of interest*

The authors declare no conflict of interest.

## Conflict of interest

The authors declare no conflict of interest.

## Figures and Tables

**Table 1:**
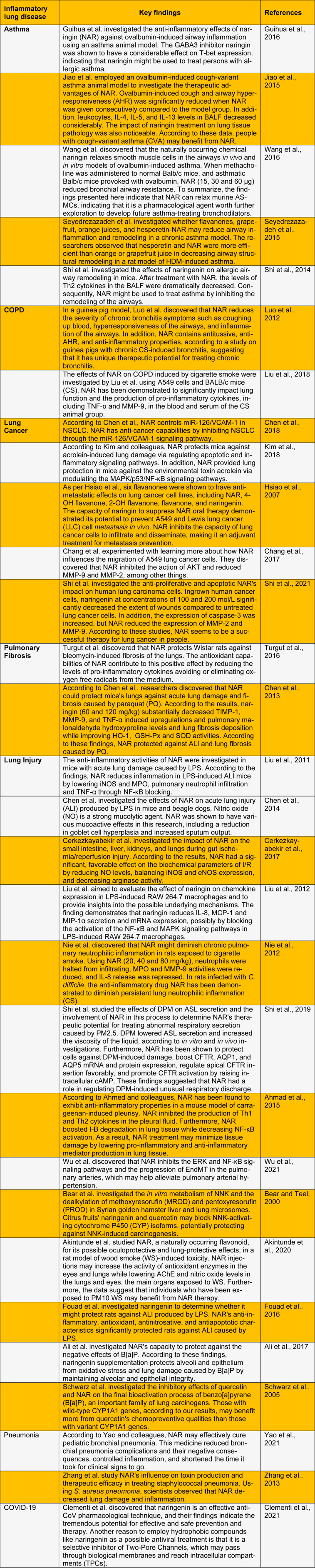
An update on the protective effect of naringin in various inflammatory lung diseases
